# Probing the mesoscopic size limit of quantum anomalous Hall insulators

**DOI:** 10.1038/s41467-022-31105-w

**Published:** 2022-07-22

**Authors:** Peng Deng, Christopher Eckberg, Peng Zhang, Gang Qiu, Eve Emmanouilidou, Gen Yin, Su Kong Chong, Lixuan Tai, Ni Ni, Kang L. Wang

**Affiliations:** 1 Department of Electrical and Computer Engineering, University of California Los Angeles, Los Angeles, CA USA; 2 Fibertek Inc, Herndon, VA USA; 3 US Army Research Laboratory, Adelphi, MD USA; 4 US Army Research Laboratory, Playa Vista, CA USA; 5 Department of Physics and Astronomy, University of California Los Angeles, Los Angeles, CA, USA

**Keywords:** Topological insulators, Phase transitions and critical phenomena

## Abstract

The inelastic scattering length (*L*_*s*_) is a length scale of fundamental importance in condensed matters due to the relationship between inelastic scattering and quantum dephasing. In quantum anomalous Hall (QAH) materials, the mesoscopic length scale *L*_*s*_ plays an instrumental role in determining transport properties. Here we examine *L*_*s*_ in three regimes of the QAH system with distinct transport behaviors: the QAH, quantum critical, and insulating regimes. Although the resistance changes by five orders of magnitude when tuning between these distinct electronic phases, scaling analyses indicate a universal *L*_*s*_ among all regimes. Finally, mesoscopic scaled devices with sizes on the order of *L*_*s*_ were fabricated, enabling the direct detection of the value of *L*_*s*_ in QAH samples. Our results unveil the fundamental length scale that governs the transport behavior of QAH materials.

## Abstract (web summary)

In quantum anomalous Hall (QAH) materials, the mesoscopic scattering length (*L*_s_) plays an instrumental role in determining transport properties. Here, the authors examine *L*_s_ in three regimes (QAH, quantum critical, and insulating) with distinct transport behaviours, and find a universal *L*_s_ across all regimes.

## Introduction

The inelastic scattering length, *L*_*s*_, is the characteristic distance that an electron travels between dephasing inelastic scattering events. It is a length scale below which quantum phase coherence is maintained at non-zero temperatures, playing a pivotal role in certain transport phenomena in condensed matters^1–8^. Above this length scale, the electrons diffuse classically, without any quantum interference effects. In this sense, while a sample can possess physical dimensions of arbitrary size, *L*_*s*_ sets a fundamental bound for the mesoscopic coherent transport, beyond which transport quantities, *e.g*. the conductance, scale with the size of the sample^2,3^. Usually, *L*_*s*_ is temperature-dependent and has the form of ${L}_{s}\left(T\right)=a{T}^{-p/2}$, where *p* is the temperature exponent^3,9,10^. The value of *p* depends on material details, such as the scattering mechanism and dimensionality of the system^4^.

As a key determiner of quantum transport phenomena, *L*_*s*_ is known to play a critical role in the quantum critical regime of quantum Hall (QH) and quantum anomalous Hall (QAH) materials. In these prototypical quantum systems, metal-insulator transitions occur when the system is tuned between adjacent Hall plateaus by the magnetic field. During the transit, the physical properties of the system have been demonstrated to depend uniquely upon the ratio between *L*_*s*_ and the diverging critical fluctuation correlation length *ξ*, as described by finite-size scaling phenomenology^11,12^. While *L*_*s*_, and particularly its power-law dependencies, have been well characterized in the critical regime for the QH systems^13,14^, a comprehensive study of *L*_*s*_ in the quantum critical regime of QAH materials is still lacking. Moreover, the systematic effort undertaken to probe *L*_*s*_ deep within the metallic^15^ and insulating phases of QAH samples has been very limited to date. Given the potentially powerful technological implications of the dissipationless transport observed in the former, and the anomalously low temperatures required for this behavior to manifest, the systematic characterization of *L*_*s*_ in the quantized regime in particular could expand the fundamental understanding of the QAH phase in such a way as to potentially guide future material improvements.

## Results

### QAH, quantum critical, and insulating regimes of the QAH insulator

**Fig. 1:**
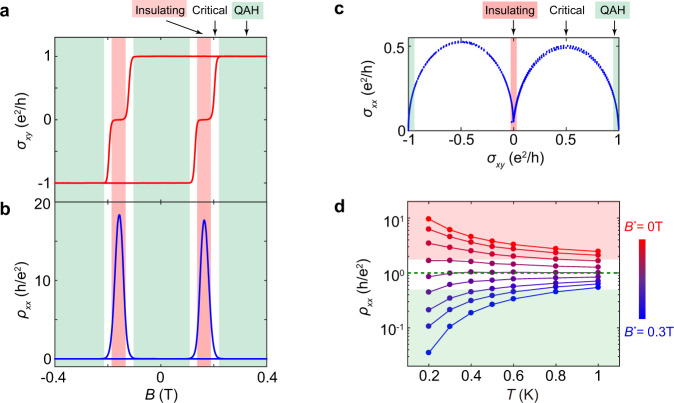
QAH, quantum critical, and insulating regimes of QAH. **a**, **b** Magnetic field dependence of *σ*_*xy*_ and *ρ*_*xx*_ measured at 50 mK. Upon field sweeping, the QAH system can be tuned into the QAH, quantum critical, or insulating regime. **c** Plot of *σ*_*xx*_ as a function of *σ*_*xy*_. A semicircle relationship is shown during the transition. **d** Temperature dependence of *ρ*_*xx*_ under different B^*^, where ${B}^{* }=B-{\mu }_{0}{H}_{c}$ and *H*_*c*_ is the coercive field, exhibiting a magnetization driven metal-insulator transition at ${B}^{* }={B}_{c}^{* }$ (dashed green line) with a critical resistance of ~*h*/*e*^*2*^.

The QAH effect was theoretically predicted and experimentally realized in Cr- and V- doped topological insulators^16–20^, and recently has also been observed in MnBi_2_Te_4_^21^ and moiré heterostructures^22,23^. By manipulating the material’s magnetization, QAH systems can be tuned into different phases, namely the QAH, quantum critical, and insulating regimes. Figure 1a, b present the field dependences of Hall conductance (*σ*_*yx*_) and longitudinal resistance (*ρ*_*xx*_), respectively, of a 6-quintuple-layer thick Cr-doped (BiSb)_2_Te_3_ sample, in which three distinct regimes can be seen. In the QAH regime where the system is fully magnetized, |*σ*_*yx*_ | exhibits a quantized value of e^2^/h. In this phase, the system is highly conductive; displaying a *ρ*_*xx*_ of only a few Ohms. That *ρ*_*xx*_ is almost vanishing indicates electrons travel with negligible dissipation in the sample. At the opposite extreme near the coercive field (*H*_*c*_) where equally populated up and down magnetic domains coexist, the system becomes highly resistive as *ρ*_*xx*_ reaches a large value (>450 kΩ). Meanwhile, *σ*_*yx*_ develops two plateaus with zero conductance, a hallmark of the insulating state^24^. The QAH and the insulating regime are bridged by the quantum critical regime, wherein *σ*_*yx*_ transitions between adjacent plateaus, as will be discussed later. During the phase transition, the conductivities follow a semicircle relation^25–28^: ${\sigma }_{{xx}}^{2}+{({\sigma }_{{xy}}-\frac{{e}^{2}}{2h})}^{2}={(\frac{{e}^{2}}{2h})}^{2}$, as shown in Fig. 1c. At the quantum critical point, *σ*_*xx*_ ~ *σ*_*yx*_ ~ 0.5 e^2^/h.

The three regimes show distinct transport behavior in the temperature dependence of *ρ*_*xx*_, as shown in Fig. 1d. In the figure, each curve was plotted under a constant B^*^, where B^*^ = *B* −*µ*_*0*_*H*_*c*_ and *B* is the external magnetic field. Here, using B^*^ instead of *B* ensures each temperature-dependent curve was taken under a fixed magnetization rather than a fixed magnetic field. As the sample’s internal magnetization is the tuning parameter that drives the QAH phase transition^24^, such a transformation assists in tracking the QAH transition over wide temperature ranges wherein the magnetic properties are also evolving. In the *T* → 0 limit, *ρ*_*xx*_ approaches zero and displays a “metallic” behavior in the QAH regime (large B^*^), while in the insulating regime (B^*^ = 0), *ρ*_*xx*_ diverges and displays an “insulating” behavior. At an intermediate field (${B}_{c}^{* }$ = 0.045 T) corresponding to the quantum critical regime, *ρ*_*xx*_ is almost temperature-independent with a critical resistance of ~ h/e^2^, a value commonly seen in the 2D metal-insulator transition^29,30^.

### The temperature exponent in different regimes

The inelastic scattering length and the temperature exponent *p* in the QAH, quantum critical, and insulating regimes, respectively, can be obtained by examining the current heating effect^3^. At low temperatures, the coupling between the electrons and phonons is very weak. In this regime, the electrons can gain additional kinetic energy when subjected to a large electric field, driving them out of thermal equilibrium with the lattice. This kinetic energy gain can be modeled as an increase in the effective temperature of the electronic system through the relationship ${k}_{B}{T}_{e} \sim {eE}{L}_{s}$, where ${eE}{L}_{s}$ is the average kinetic energy accumulated by electrons between relaxation events and ${k}_{B}{T}_{e}$ is the effective thermal energy it corresponds to. Since *L*_*s*_ depends on temperature, ${L}_{s} \sim {T}_{e}^{-p/2}$, the effective temperature would have a power-law relationship with the applied field (or the voltage) as ${T}_{e} \sim {E}^{2/(2+p)}( \sim {V}^{2/(2+p)})$. The value of *p* can thus be obtained by carefully monitoring the electric field dependence of *T*_*e*_. In the quantum critical, QAH, and insulating regimes, this is accomplished by measuring the maximum slope of the Hall conductance ${(\partial {\sigma }_{{xy}}/\partial B)}_{{\max }}$, the residual resistance of *ρ*_*xx*_, and the longitudinal resistance peak (*ρ*_*xx*_^*peak*^), respectively, as discussed below.

In the quantum critical regime, the QAH system experiences a metal-insulator transition as *σ*_*xy*_ transitions between adjacent plateaus^26,28,31,32^. According to the finite-size scaling ansatz^11,12,33,34^, the maximum slope of *σ*_*xy*_, ${(\partial {\sigma }_{{xy}}/\partial B)}_{{\max }}$ during the transition, exhibits a power-law scaling relationship with the temperature as ${(\partial {\sigma }_{{xy}}/\partial B)}_{{\max }} \sim {T}^{-\kappa }$, where *κ* is the temperature scaling critical exponent. When held at a fixed temperature, ${(\partial {\sigma }_{{xy}}/\partial B)}_{{\max }}$ also displays a scaling relationship with the current as ${(\partial {\sigma }_{{xy}}/\partial B)}_{{\max }} \sim {I}^{-b}$, where *b* is the current scaling critical exponent^9,11,12^. These exponents *κ* and *b* are themselves convolutions of correlation length exponent *ν* and temperature exponent *p*, satisfying the relationships^35,36^: *κ* = *p*/2*ν* and *b* = *p*/(*p*+2)*ν*, thus the value of *p* can be obtained via experimentally measured values of *κ* and *b*.

**Fig. 2:**
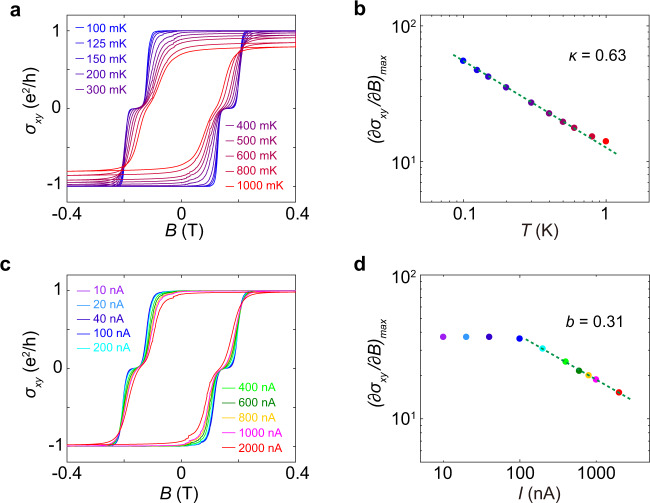
Scaling behaviors and critical exponents in the quantum critical regime. **a** Field dependence of *σ*_*xy*_ at different temperatures. All curves are measured using a 10 nA excitation current. **b** Temperature dependence of ${{{\mbox{(}}}\partial {\sigma }_{{xy}}{{\mbox{/}}}\partial B{{\mbox{)}}}}_{{\max }}$, demonstrating a temperature scaling relationship as ${{{\mbox{(}}}\partial {\sigma }_{{xy}}{{\mbox{/}}}\partial B{{\mbox{)}}}}_{{\max }} \sim {T}^{-\kappa }$. **c** Field dependence of *σ*_*xy*_ at different currents. All curves are measured at 200 mK. **d** Current dependence of ${{{\mbox{(}}}\partial {\sigma }_{{xy}}{{\mbox{/}}}\partial B{{\mbox{)}}}}_{{\max }}$, demonstrating a current scaling relationship as ${{{\mbox{(}}}\partial {\sigma }_{{xy}}{{\mbox{/}}}\partial B{{\mbox{)}}}}_{{\max }} \sim {I}^{-b}$ under large currents. The saturation of ${{{\mbox{(}}}\partial {\sigma }_{{xy}}{{\mbox{/}}}\partial B{{\mbox{)}}}}_{{\max }}$ at *I* = 100 nA indicates the effective tem*p*erature is comparable to the bath temperature at this current. From the scaling relationship ${{{\mbox{(}}}\partial {\sigma }_{{xy}}{{\mbox{/}}}\partial B{{\mbox{)}}}}_{{\max }} \sim {T}^{-\kappa } \sim {I}^{-b}$, we have *p* = 2.06.

Figure 2a presents the field dependence of *σ*_*xy*_ for varying temperatures, showing that the transitions between *σ*_*xy*_ plateaus broaden as the temperature increases. Plotted in a log-log plot (Fig. 2b), ${(\partial {\sigma }_{{xy}}/\partial B)}_{{\max }}$ displays a linear relationship with *T*, indicating the predicted scaling relationship ${(\partial {\sigma }_{{xy}}/\partial B)}_{{\max }} \sim {T}^{-\kappa }$. From the linear fit of the slope, we extracted the exponent *κ* = 0.63 ± 0.01. Meanwhile, Fig. 2c presents the field dependence of *σ*_*xy*_ for different currents. Fitting these data we find ${(\partial {\sigma }_{{xy}}/\partial B)}_{{\max }} \sim {I}^{-b}$, wherein *b* = 0.31 ± 0.01 (Fig. 2d). Note that ${(\partial {\sigma }_{{xy}}/\partial B)}_{{\max }}$ saturates at small currents. In this saturated regime, the effective temperature due to the current heating is lower than the bath temperature. Combining the temperature and current scaling results, we find *p* = 2.06 ± 0.14.

**Fig. 3:**
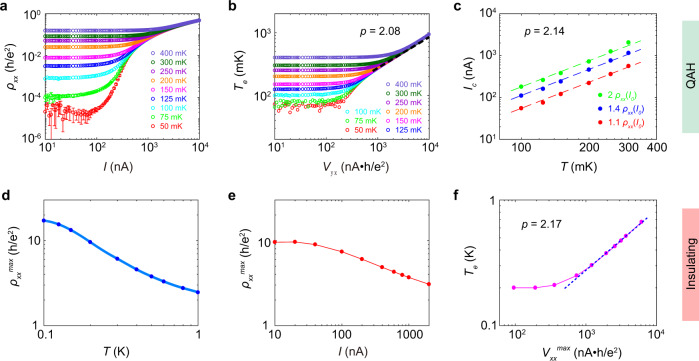
Critical exponent in the QAH and insulating regimes. **a** Current dependence of *ρ*_*xx*_ at various temperatures. All data are measured under a 1 T magnetic field. **b** Effective temperature *T*_*e*_ vs. Hall voltage *V*_*yx*_. Under small currents, *T*_*e*_ equals the bath temperature. As the current increases, the electrons are dominantly thermalized by the electric field, and all data approach a universal curve (fitted by the black dashed lines) with a slope of 2/(*p*+2) = 0.49, yielding *p* = 2.08 in the QAH regime. **c** Temperature dependence of *I*_*c*_. The breakdown current at a given temperature is defined as the current at which *ρ*_*xx*_ exceeds *m* times of *ρ*_*xx*_ measured at the base current, where *m* = 1.1 (red), 1.4 (blue), or 2 (green). The slope of the *I*_*c*_ vs. *T* curve in the log-log plot gives *p* = 2.14. **d** Temperature dependence of *ρ*_*xx*_^*max*^. The blue dots are obtained from the resistance maximum in the field-dependent *ρ*_*xx*_ curves measured at different temperatures and the cyan lines are extrapolated from the blue dots. **e** Current dependence of *ρ*_*xx*_^*max*^. **f** Effective temperature *T*_*e*_ vs. voltage *V*_*xx*_^*max*^, under large currents the slope in the log-log plot is 2/(*p*+2) = 0.48, yielding *p* = 2.17 in the insulating regime.

In the QAH regime where the system is fully magnetized, the longitudinal resistance is exceptionally small and the Hall resistance is quantized. Therefore, a current sourced along the longitudinal direction will produce an electric field predominantly along the transverse direction $E\approx {E}_{y} \sim {V}_{{yx}}/W={Ih}/{e}^{2}W$, where *E*_*y*_ and *V*_*yx*_ are the Hall electric field and Hall voltage, respectively, *I* is the applied current, and *W* is the width of the Hall-bar device. Figure 3a presents the current dependence of *ρ*_*xx*_ under different temperatures (the same plot for *ρ*_*yx*_ is shown in Supplementary Fig. 2). For all temperatures, *ρ*_*xx*_ maintains a constant value for relatively small currents. Once the current exceeds a temperature-dependent critical value, however, *ρ*_*xx*_ begins to increase, indicating that the electrons are heated above the bath temperature by the large electric field. We also measure the temperature dependence of *ρ*_*xx*_, as shown in Supplementary Fig. 3, where a small current of 10 nA is applied in order to minimize the current heating effect. By mapping the *I* (or *V*_*yx*_)-dependent *ρ*_*xx*_ curve to the *T*-dependent *ρ*_*xx*_ curve, the effective temperature plot as a function of *V*_*yx*_ can be obtained as shown in Fig. 3b. The linear relationship in the log-log plot at high currents indicates the power-law relationship between *T*_*e*_ and *V*_*yx*_: ${T}_{e} \sim {{V}_{{yx}}}^{2/(2+p)}$. From the linear fit of the slope, the value of the temperature exponent *p* is determined to be *p* = 2.08 ± 0.03.

The temperature exponent in the QAH regime can also be obtained from the breakdown current (*I*_*c*_). For each temperature, we define the current at which *ρ*_*xx*_ exceeds *m×ρ*_*xx*_(*I*_*0*_) as the breakdown current *I*_*c*_(*T*), where *I*_*0* _= 10 nA and *m* is varied to demonstrate that the physics presented is independent of the precise definition of the breakdown condition. That *ρ*_*xx*_ starts to deviate from *ρ*_*xx*_(*I*_*0*_) at *I*_*c*_ indicates the effective temperature is comparable to the bath temperature under this current. Figure 3c presents the temperature dependence of *I*_*c*_ for *m* = 1.1, 1.4, and 2. As can be seen, *I*_*c*_ displays a linear relationship with *T* in the log-log plot. Since ${I}_{c} \sim {V}_{{yx}} \sim {T}^{(p+2)/2}$, we have *p* = 2.14 ± 0.02 for all *m*. Notably, the *p* value obtained from breakdown currents is close to that obtained from high current results discussed above. Previous studies attribute the mechanism of the current breakdown in QAH samples to the field-assisted variable range hopping or bootstrap electron heating^15,37^. Here the results show that the breakdown of QAH under large current shares the same origin as the breakdown against thermal excitation. Therefore, we anticipate that achieving QAH with a higher quantization temperature would also improve the maximum current they can sustain, which is of great practical use in the application of dissipationless transport.

Finally, in the insulating regime where the QAH sample is at its coercive field, the topological edge transport is suppressed. However, percolative longitudinal transport still occurs^24^, displaying an insulating temperature dependence. By tracking the temperature and current dependencies of this maximal resistance, the critical exponent *p* can be extracted in this insulating phase. At the coercive field, since *ρ*_*xx*_ is large and *ρ*_*yx*_ becomes zero, the electric field lies predominantly along the longitudinal direction, *i.e*., $E\approx {E}_{x} \sim {V}_{{xx}}=I{{\rho }_{{xx}}}^{{\max }}$, where *V*_*xx*_ is the voltage drop along the longitudinal direction. The temperature dependence of *ρ*_*xx*_^*max*^ is obtained by extracting the maximum value in the field-dependent *ρ*_*xx*_ isotherms measured with small excitation currents (Supplementary Fig. 4a). As can be seen in Fig. 3d, *ρ*_*xx*_^*max*^ displays a monotonic decrease with increasing temperature. Likewise, from the field dependence of *ρ*_*xx*_ measured under different currents (Supplementary Fig. 4b), the current dependence of *ρ*_*xx*_^*max*^ can be obtained, as shown in Fig. 3e. By comparing the data points in the *ρ*_*xx*_^*max*^ vs. *I* curve and the *ρ*_*xx*_^*max*^ vs. *T* curve, each voltage drop at *ρ*_*xx*_^*max*^, *V*_*xx*_^*max*^, can be one-to-one mapped to an effective temperature *T*_*e*_, as shown in Fig. 3f. Under high voltages, *T*_*e*_ displays a linear relationship with *V*_*xx*_^*max*^ in the log-log plot, the slope of which gives a temperature exponent value of *p* = 2.17 ± 0.02 in this insulating regime.

Despite the dramatic change in conduction behaviors across the different field-driven phases of the QAH material, the value of *p* shows strikingly little variation; with observed values of 2.06, 2.14, and 2.17 in the critical, QAH, and insulating regimes respectively. As the value of *p* describes the reduction of the inelastic scattering length with temperature, and generally reflects the dominant excitations from which electrons scatter, this observation indicates the electrons interact with their environment identically in all transport regimes. Such a result indicates a universal transport behavior in the QAH system, and supports theoretical suggestions that a single Hamiltonian can describe the QAH system throughout the plateau-plateau transition^38^. We would like to note, however, that different values for *p* are commonly reported in different QH systems, where variations are commonly attributed to differences in the quenched disorders hosted by the materials^13,39,40^. As such, we do not expect this value of *p* to necessarily be universal across different QAH samples. On the other hand, a universal value of *p* is revealed within individual QAH samples, due to the fact that the quenched disorder remains fixed when samples are tuned across different conduction regimes. We further note that the value of *p* observed here is close to the value of *p* = 2 that is commonly reported for QHE materials dominated by electron–electron scattering, suggesting a similar mechanism may dominate these QAH samples^14,35,41–46^.

### Estimation of the inelastic scattering length in different regimes

In an effort to further characterize inelastic scattering in the QAH system, we now analyze the absolute value of *L*_*s*_ in the critical, QAH, and insulating regimes. As *p* is determined for these regimes, obtaining *L*_*s*_ at any given temperature, *e.g*., 200 mK, would disclose *L*_*s*_ at all temperatures via the relationship ${L}_{s}\left(T\right)=a{T}^{-p/2}$. The effective temperature resulting from the current heating effect can be expressed as^3^ $\pi {k}_{B}{T}_{e}=4{eE}{L}_{s}$. Therefore, the inelastic scattering length can be estimated if the electric field at this temperature is known for three regimes, as illustrated below.

1. In the quantum critical regime, both *ρ*_*xx*_ and *ρ*_*yx*_ are exhibit values close to the resistance quantum ~ h/e^2^, therefore the electric field has components along both longitudinal and transverse directions with ${E}_{y}=I{\rho }_{{yx}}/W$ and ${E}_{x}=I{\rho }_{{xx}}/W$, wherein the width of the Hall-bar *W* = 0.5 mm. As shown in Fig. 2d, the increasing trend of ${(\partial {\sigma }_{{xy}}/\partial B)}_{{\max }}$ with decreasing current saturates at 100 nA, indicating the effective temperature is comparable to the bath temperature 200 mK at this current, giving *L*_*s*_ ~ 1.85 μm in this regime.
2. In the QAH regime, $E\approx {E}_{y}=I{\rho }_{{yx}}/W$. As discussed above, the effective temperature is comparable to the bath temperature when *I* = *I*_*c*_. It can be seen in Fig. 3c, at 200 mK, *I*_*c*_ = 220 nA (for m = 1.1), therefore, *L*_*s*_ is estimated to be ~1.19 μm in the QAH regime.
3. In the insulating regime at the coercive field, *ρ*_*xx*_ is large and *ρ*_*yx*_ is around zero. Therefore, $E\approx {E}_{x}={{V}_{{xx}}}^{{\max }}/L$ and *L* is the effective length of the Hall-bar devices. As evidenced in Fig. 3f, the effective temperature is about 200 mK when *V*_*xx*_^*max*^ = 192.5 nA·h/e^2^. Thus, the estimated *L*_*s*_ is ~ 1.36 μm in the insulating regime.

The above results show that the value of *L*_*s*_ in these three distinct regimes not only share a universal temperature exponent *p* but also have the same order of magnitude at 200 mK, despite the 5 orders of magnitude change in resistance and the transition from “metallic” to “insulating” temperature dependences between the different transport regimes. The implication of the comparable values of *L*_*s*_ and *p* obtained in different regimes is twofold. There are two kinds of disorder in QAH materials, one is the quenched disorder (crystalline defects, Cr doping disorder, Bi-Sb alloying disorder, etc.) and the other is the disorder from the magnetic domain structure. When the system is in the QAH regime, the magnetism is in a single domain, whereas the system features an even population of randomly distributed up and down magnetic domains in the insulating regime^47^. Thus, while the quenched disorder is fixed, disorder from magnetic domains changes from maximally ordered to maximally disordered when the system is tuned from the QAH to the insulating regime. Since *L*_*s*_ is unchanged during the phase transition, the results above clearly indicate that the dominant inelastic scattering occurs from quenched disorder, with no apparent contribution from the disordered magnetic domain structure in the insulating phase. Meanwhile, the observation of the constant critical exponent *p* indicates the nature of the interaction between electrons and these disorders is unchanged through the quantum phase transition.

### Direct detection of *L*_*s*_ in the mesoscopic scaled QAH device

**Fig. 4:**
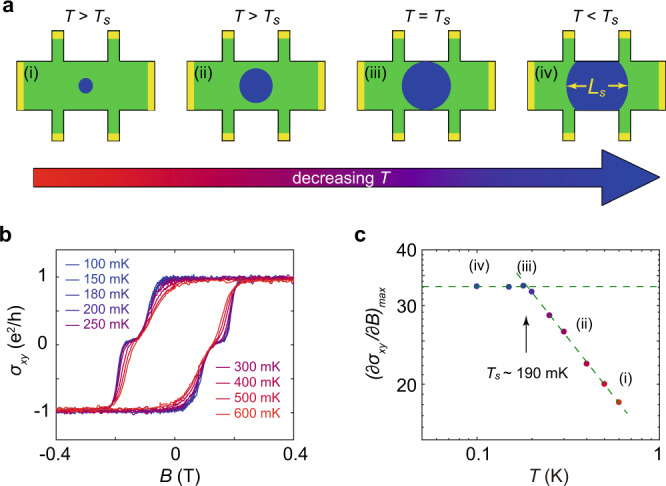
Saturation of (*∂σ*_*xy*_/*∂B*)_*max*_ in the quantum critical regime at low temperatures for a 5-μm QAH device. **a** Illustration of *L*_*s*_ at different temperatures, green regions represent the area of the Hall-bar device and blue regions indicate the length scale of *L*_*s*_. From scenario_*s*_ (i) to (iii), *L*_*s*_ grows as the temperature is lowered. In _*s*_cenario (iii), *L*_*s*_ is comparable to the physical size of the Hall-bar device. In scenario (iv), *L*_*s*_ is limited by the physical size. **b** Field dependence of *σ*_*xy*_ at different temperatures, all curves were measured with a 0.5 nA current. **c** Temperature dependence of ${{{\mbox{(}}}\partial {\sigma }_{{xy}}{{\mbox{/}}}\partial B{{\mbox{)}}}}_{{\max }}$ in the quantum critical regime. The scaling behavior saturates at ~190 mK, indicating *L*_*s*_ reaches the physical size of the sample at this temperature. (i)-(iv) correspond to the scenarios illustrated in **a**.

While the above approximations of *L*_*s*_ are relatively crude in nature, they notably suggest that standard lithographic processes may be used to fabricate devices with feature sizes comparable to the characteristic length scale of the material. For samples with a large enough size, during the phase transition, the observable quantities of the system are solely determined by the ratio of *L*_*s*_ and the correlation length of order parameter fluctuations *ξ*^11,12^. However, in small devices measured at sufficiently low temperatures, the material’s natural inelastic scattering length may become comparable to the physical size of the sample *L*, artificially bounding the effective value of *L*_*s*_ experienced by electrons in the device. As a consequence of this bounding, a temperature *T*_*s*_ will exist such that the ratio *L*_*s*_/*ξ*, and consequentially the critical transport of the sample, will remain constant for all temperatures below *T*_*s*_. Here *T*_*s*_ represents the point for which *L*_*s*_ = *L*, enabling an exact, experimental determination of *L*_*s*_ at this temperature (Fig. 4a).

To experimentally observe such an effect, we fabricated Hall bars with a size of 5 μm × 15 μm using e-beam lithography. Figure 4b presents the field dependence of *σ*_*xy*_ for the 5-μm-sample at different temperatures, and Fig. 4c shows the temperature dependence of ${(\partial {\sigma }_{{xy}}/\partial B)}_{{\max }}$ at the quantum critical regime. Instead of the diverging increase with decreasing temperature as shown for the large sample in Fig. 2b, ${(\partial {\sigma }_{{xy}}/\partial B)}_{{\max }}$ saturates abruptly at around 190 mK. This indicates *L*_*s*_ reaches the physical size of the sample at this temperature. That *L*_*s*_ equals 5 μm at 190 mK is in good agreement with the estimation obtained from the current heating results. Note that in order to avoid the saturation caused by the current heating, a current as small as 0.5 nA was used for the measurement. To further rule out such a heating effect, we use different currents to measure at 100 mK. As can be seen in Supplementary Fig. 6, the value of ${(\partial {\sigma }_{{xy}}/\partial B)}_{{\max }}$ remains constant when the current does not exceed 0.5 nA, proving the current heating effect negligible in the experiment. As a confirmation that this effect is reflective of the intrinsic finite-size effect, the measurement has been repeated in another 5-μm-device shown in Supplementary Fig. 7. The finite-size effect is of practical importance for the application of QAH-based electronic devices. While it is broadly desirable to fabricate more compact devices, for a coherent quantum system like QAH, the finite size limits its coherence at low temperature, significantly impacting the performance of the device. As such, special care must be taken in choosing the proper size of the devices.

## Discussion

To summarize, we obtained *L*_*s*_ in different conduction regimes of the QAH by analyzing the current heating results. A temperature exponent *p* ~ 2.1 and a value on the order of 1 μm for *L*_*s*_ at 200 mK were found in all regimes, revealing a universal inelastic scattering length in QAH. Furthermore, in mesoscopic devices, we observed the finite-size effect as *L*_*s*_ reaches the physical size of the device at low temperatures, enabling the direct detection of *L*_*s*_ in QAH. Our work unveils the fundamental length scale in QAH samples, which sheds light on the understanding of mesoscopic transport in QAH materials and facilitates the implementation of dissipationless electronics in QAH-based devices.

## Methods

### Sample growth

The molecular beam epitaxy grown samples were prepared in a Perkin-Elmer chamber with a base vacuum of $5\times {10}^{-10}$ Torr. High-purity Cr(99.995%), Bi(99.999%), Sb(99.999%), and Te(99.9999%) were deposited on the epi-ready semi-insulating GaAs(111)B substrates. The growth process was monitored using in-situ Reflection high-energy electron diffraction.

### Device fabrication

Grown films were patterned into Hall bars for transport measurements. The 0.5-mm-devices were fabricated using hard masks and reactive-ion etching. The 5-μm mesoscopic devices were patterned by the e-beam lithography.

### Transport measurements

Transport measurements were carried out on a Physical Property Measurement System (Quantum Design) with a dilution refrigerator insert (50 mK, 9 T).

## Supplementary information

Supplementary Information

## Data Availability

All data for the figures and other Supplementary information that support this work are available upon reasonable request to the corresponding author.
