# Supplementary material for: Probing the mesoscopic size limit of quantum anomalous Hall insulators
Source: Nat Commun. 2022 Jul 22;13:4246. doi: 10.1038/s41467-022-31105-w (PMC9307791; doi:10.1038/s41467-022-31105-w)
Supplement: Supplementary file 1 — Supplementary Information [file 41467_2022_31105_MOESM1_ESM.pdf]

Supplementary Information

Probing the mesoscopic size limit of quantum anomalous Hall insulators

Peng Deng[1*], Christopher Eckberg[2,3,4*], Peng Zhang[1], Gang Qiu[1], Eve Emmanouilidou[5], Gen Yin[1], Su Kong Chong[1], Lixuan Tai[1], Ni Ni[5], and Kang L. Wang[1,5*]

[1]Department of Electrical and Computer Engineering, University of California Los Angeles, Los Angeles, California 90095, USA

[2] Fibertek Inc., Herndon, VA 20171

[3] US Army Research Laboratory, Adelphi, MD 20783

[4]US Army Research Laboratory, Playa Vista, CA 90094

[5]Department of Physics and Astronomy, University of California Los Angeles, Los Angeles, California 90095, USA

[*]Corresponding authors: dengpeng@g.ucla.edu (P. D.), chris.eckberg@fibertek.com (C. E.), wang@ee.ucla.edu (K. L. W.)

1. Transport behavior in the $T \rightarrow 0$ limit.

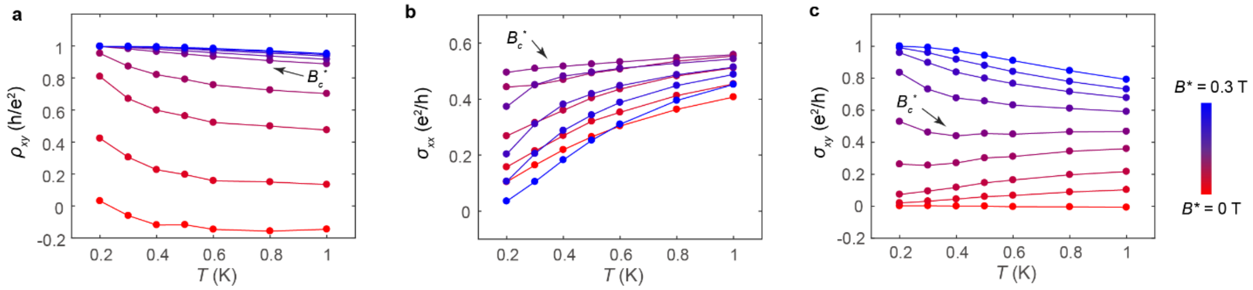

Supplementary Figure 1. The limiting transport behavior of ρ_{yx}, σ_{xy}, and σ_{xy} in the $T \rightarrow 0$ limit under different B^*. The red, purple (denoted by B_c^*), and blue data represent the QAH, quantum

critical, and insulating regimes, respectively.

2. Current dependence of ρ_{yx} in the QAH regime.

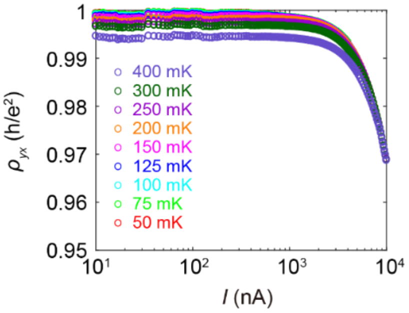

Supplementary Figure 2. Current dependence of ρ_{yx} under different temperatures in the QAH regime. All data are measured under a 1 T magnetic field.

3. Temperature dependence of ρ_{xx} in the QAH regime.

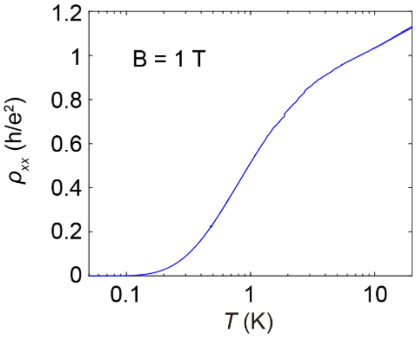

Supplementary Figure 3. Temperature dependence of ρ_{xx} under 1 T magnetic field. The curve was measured using a 10 nA excitation current.

4. Field dependence of ρ_{xx}.

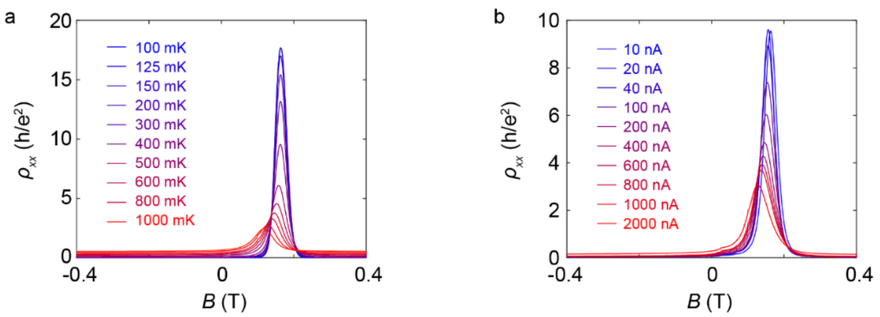

Supplementary Figure 4. a, Field dependence of ρ_{xx} under different temperatures. The field sweeping direction is from negative to positive. All curves are measured under a 10 nA current. **b,** Field dependence of ρ_{xx} under different currents. All curves are measured at 200 mK.

5. Inelastic scattering exponent *p* in another sample

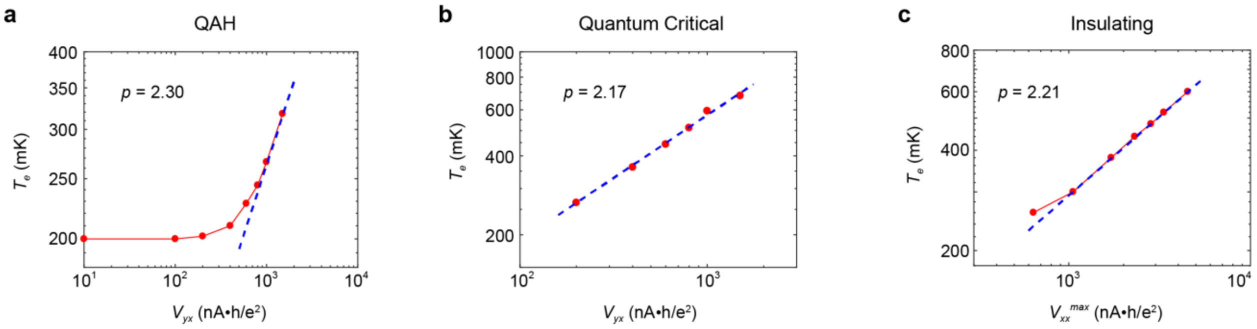

Supplementary Figure 5. Inelastic scattering exponent p obtained in the **a**, QAH, **b**, quantum critical, and **c**, insulating regimes. Although the values are slightly larger than that examined in the main text, the values are comparable for different regimes in this sample.

6. Current dependence of $(\partial\sigma_{xy}/\partial B)_{max}$ in the quantum critical regime for the 5-μm-sample

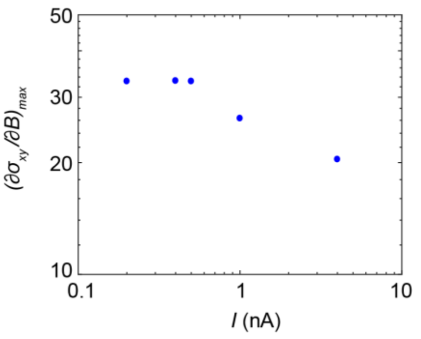

Supplementary Figure 6. Current dependence of $(\partial\sigma_{xy}/\partial B)_{max}$. All data are measured at 100 mK. The current heating effect is neglectable when I is no larger than 0.5 nA.

7. Finite-size effect in another 5-μm-sample.

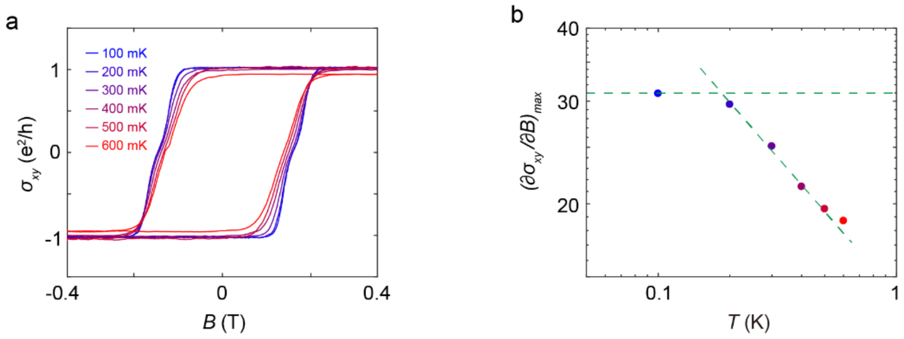

Supplementary Figure 7. a, Field dependence of σ_{xy} at different temperatures. **b,** Temperature dependence of $(\partial\sigma_{xy}/\partial B)_{max}$. For this sample, the saturation temperature is also around 190 mK.
